# Quantifying molecular aggregation by super resolution microscopy within an excitatory synapse from mouse hippocampal neurons

**DOI:** 10.1016/j.xpro.2021.100470

**Published:** 2021-04-14

**Authors:** Shekhar Kedia, Narendrakumar Ramanan, Deepak Nair

**Affiliations:** 1 Centre for Neuroscience, Indian Institute of Science, Bangalore 560012, India

**Keywords:** Cell culture, Microscopy, Neuroscience

## Abstract

Super-resolution microscopy (SRM) has been widely adopted to probe molecular distribution at excitatory synapses. We present an SRM paradigm to evaluate the nanoscale organization heterogeneity between neuronal subcompartments. Using mouse hippocampal neurons, we describe the identification of the morphological characteristics of nanodomains within functional zones of a single excitatory synapse. This information can be used to correlate structure and function at molecular resolution in single synapses. The protocol can be applied to immunocytochemical/histochemical samples across different imaging paradigms.

For complete details on the use and execution of this protocol, please refer to Kedia et al. (2021).

## Highlights

• Functional zones within synapses of mouse hippocampal neurons can be identified by SRM
• Combination of segmentation and morphometry yield variability of nanodomains
• Nanoscale morphology of molecular organization is heterogeneous within synapses

## Graphical Abstract

**Figure:**
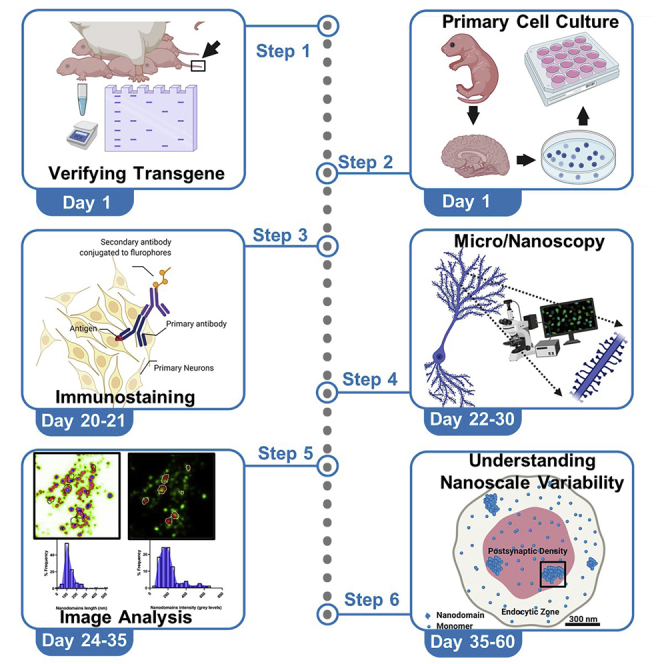

## Before you begin

The protocol defines detailed steps involved in the evaluation of molecular segregation of proteins of interest within functional zones of individual excitatory synapses. The following paradigm uses diffraction limited images obtained from confocal microscopy and ensemble based super resolution images obtained by stimulated emission depletion microscopy. We describe the immunostaining of primary mice hippocampal neurons and optimal parameters that were used for concomitant confocal and ensemble-based super resolution imaging. We illustrate the segmentation protocol to extract selected regions of interest within neuronal sub compartments to analyze the quantitative differences in the nanoscale association patterns of molecules that are heterogeneously clustered in various functional zones of the synapse. Though the method describes the evaluation of nanoscale heterogeneity in primary neuronal cell cultures, it could also be adapted for adherent non-neuronal cells. Additionally, it can be opted for a generalized evaluation of images obtained through widefield microscopy and single molecule based super resolution microscopy and immunohistochemical samples imaged through both diffraction limited and super resolution paradigms.

### Immunocytochemistry

**Timing: ∼4 h**

1. Take mixed sex primary hippocampal neuronal culture from post-natal day 0 or 1 (P0-P1) wild type C57BL/6 mice.

2. If hippocampal neuronal culture is desired from transgenic mice models for Alzheimer’s disease such as APPswe/PS1ΔE9 transgenic mice, perform genotyping for APP-Swe transgene as follows:
  a. Cut 1 to 2 mm of mouse tail with a clean scissor and transfer to a 0.6 mL polypropylene microcentrifuge tube.
    ***Note:*** The cut tail sample can be stored at −20^°^C for 1 month.
  b. Add 75 mL of 1× tail lysis buffer.
  c. Heat at 95^°^C for 1 h.
  d. Cool sample to 10^°^C.
  e. Add 75 mL of 1× tail neutralization buffer to the tube and mix well.
    **CRITICAL:** Do not centrifuge.
  f. Take 1–2 μL of the tail digest for PCR.
  g. The PCR conditions are as follows:

**Table:** 

PCR cycling conditions			
Steps	Temperature	Time	Cycles
Initial Denaturation	95^°^C	3 min	1
Denaturation	95^°^C	30 sec	30–35 cycles
Annealing	55^°^C	15 sec	
Extension	72^°^C	30 sec	
Final Extension	72^°^C	10 min	1
Hold	4^°^C	forever
  h. Run reaction on a 1% agarose gel.
    ***Note:*** The prepared tail samples can be stored at −20^°^C for up to a month.
    ***Note:*** All experiments involving animals were carried out in accordance with institutional guidelines for the use and care of animals after approval from the Institutional Animal Ethics Committee (IAEC), Indian Institute of Science, Bangalore, India.

3. Seed cells at a density of 0.1×10^6^ cells/mL in 18 mm glass coverslips (Marienfeld) in a 12-well cell culture plate.

***Note:*** Primary hippocampal neuronal cultures can be established and maintained according to a previously published protocol (Beaudoin et al., 2012).

4. Use DIV 20–21 healthy primary hippocampal neurons for immunocytochemical evaluation.
5. Fix neuronal cells by incubating for 10 min at 4^°^C with the fixative solution.

***Note:*** 500 μL of 4% paraformaldehyde (PFA) plus 4% sucrose prepared in PBS per coverslip can be used as a fixative solution. We have used PFA as a fixative of choice if proteins to be immunocytochemically analyzed are either markers for functional zones of an excitatory synapse or are proteins involved in the amyloidogenic pathway like APP or Secretases. However, in some cases, PFA can also mask the recognition of specific epitope by the primary antibodies. An alternative fixative solution like ice-cold methanol could be used for 5–7 min in such cases.

**CRITICAL:** Do not inhale paraformaldehyde and prevent exposure to eyes and skin as paraformaldehyde is a moderately toxic chemical. It is also a confirmed human carcinogen.

6. Aspirate the fixative and wash cells with PBS (3 times, quick wash) at 26^°^C–28^°^C.

***Note:*** Perform all the subsequent steps at 26^°^C–28^°^C unless otherwise indicated.

**Pause point:** At this point, if required, the experiment can be halted, and fixed neurons can be stored in PBS at 4^°^C.

***Optional:*** If non-specific background fluorescence intensity is strong (see Troubleshooting), perform quenching of unreacted aldehydes after step 6 by washing with 0.1 M glycine followed by washing cells with PBS (3 times, quick wash).

7. Permeabilize cells with 0.25% Triton X-100 for 5 min.

**CRITICAL:** The permeabilization in step 7 should be avoided if the aim is to stain only surface proteins.

***Alternatives:*** Several alternative reagents for cell permeabilization are available like Tween 20, Saponin, Digitonin etc. These reagents perforate the membrane differentially and to different extents. Therefore, the suitability of the reagent depends on proteins of interest and should be determined empirically.

8. Wash cells with PBS (3 times, quick wash).
9. Incubate with 10% BSA prepared in PBS for 30 min for blocking non-specific binding sites of antibodies.
10. Aspirate the blocking solution and incubate cells with appropriately diluted primary antibodies (APP, 1:500 dilution, PSD95, 1:500 dilution for co-staining APP and postsynapses in one set of neuronal coverslips and PSD95, 1:500 dilution, Shank2, 1:500 dilution for co-labeling two excitatory postsynaptic proteins in second set of coverslips) for 2 h in a humidified chamber. Dilute primary antibody with 3% BSA prepared in PBS.

***Note:*** Parafilm can be mounted on the bottom surface of the petri plates, and the chamber can be humidified by placing tissue paper soaked in distilled water. Diluted primary antibodies can be placed on the parafilm, and the coverslips should be inverted, i.e., cells facing down. Then inverted coverslips should be placed over the antibody solution on the parafilm. This technique conserves antibody solution and requires as little as 80 μL per 18 mm coverslip for uniformly coating the coverslip surface. In contrast, the alternate methods like adding the antibody solution directly to each well of a 12-well cell culture plate requires the addition of solution as large as 300–400 μL.

***Note:*** In cases where co-immunostaining is desired for multiple proteins of interest, a cocktail of primary antibody solution can be prepared by adding the required volume of each primary antibody to 3% BSA prepared in PBS.

***Optional:*** In some instances, if the observed fluorescence intensity of the immunostained sample is low (see Troubleshooting), primary antibody incubation can also be performed for 12–14 h in a humidified chamber at 4^°^C.

11. Transfer coverslips to wells of a 12-well plate. Wash cells with 3% BSA prepared in PBS (4 times, 3 min each).

**CRITICAL:** Precaution should be taken while transferring the coverslips back to a 12-well plate. Cells should now face upwards.

12. Aspirate the washing solution and incubate cells with appropriately diluted species-specific secondary antibodies conjugated with a fluorescent dye (Alexa Fluor 594, 1:400 dilution, Abberior star red, 1:400 dilution for revealing APP co-stained with PSD95 in one set of neuronal coverslips and Alexa Fluor 488, 1:400 dilution, Alexa Fluor 555, 1:400 dilution for visualizing neurons counterstained for PSD95 and Shank2 in the second set of coverslips) for 45 min in a humidified chamber in the dark. Dilute secondary antibody with 3% BSA prepared in PBS.

***Note:*** Similar to step 10, diluted secondary antibodies can be placed on the parafilm, and the coverslips should be inverted, i.e., cells facing down. Then inverted coverslips should be placed over the antibody solution on the parafilm. This technique requires antibody solution as little as 80 μL per 18 mm coverslip.

***Note:*** Similar to step 10, a cocktail of secondary antibody solution can be prepared by adding the required volume of each secondary antibody to 3% BSA prepared in PBS.

**CRITICAL:** The incubation with fluorescent dye conjugated secondary antibodies should be performed in the dark. Aluminium foil can also be used to cover petri plates during secondary antibody incubation.

**CRITICAL:** For STED imaging with STED inverted microscope (Abberior Expert Line 775 nm, Abberior Instruments GmbH, Göttingen, Germany), we have tested the suitability of Alexa Fluor 594, Alexa Fluor 555 and Abberior star red dyes. While Alexa Fluor 647 did not give the desired contrast and resolution improvement.

***Alternatives:*** Many other dyes would be appropriate for confocal and STED imaging which should be empirically determined by the user.

13. Transfer coverslips to wells of a 12-well plate. Wash cells with PBS (4 times, 3 min each).

**CRITICAL:** Similar to step 11, precaution should be taken while transferring the coverslips back to a 12-well plate. Cells should now face upwards.

**CRITICAL:** The 12-well plate should be kept in the dark for incubation at 26^°^C–28^°^C during washing step 13. Aluminium foil can also be used to cover 12-well plate during incubation time for washing cells.

***Note:*** Staining multiple proteins simultaneously works well for synaptic proteins or proteins involved in the amyloidogenic pathway like APP or Secretases. However, for other proteins, the choice between sequential (repeat steps 9–13 for the second round of staining with a different primary and secondary antibody) and simultaneous staining should be empirically determined.

***Note:*** For staining both surface as well as cytoplasmic proteins, sequential labeling should be performed. At first, the surface proteins should be labelled by following the steps 1–13 (skipping the permeabilization in step 7). These steps will label the surface proteins. For the next round of labeling for cytoplasmic proteins, follow the steps 7–13 (including the permeabilization in step 7). These steps will label the cytoplasmic proteins. Following this procedure is especially crucial, if the aim of the experiment is to visualize the nanoscale distribution of the proteins of interest like extracellular part of APP in subsynaptic functional zones of the synapse.

14. Prepare microscope slides and mount coverslips using a drop of Prolong antifade mounting medium for confocal or STED imaging. Cells should now face downwards.

***Alternatives:*** Alternate antifade mounting medium with or without DAPI can also be used.

15. Incubate the sample for 24 h in the dark at 26^°^C–28^°^C. Cover slide box with aluminium foil as well to minimize the exposure to light.
16. Store samples in the dark at 4^°^C.

### Stimulated emission depletion microscopy (STED)

**Timing: 24 h after mounting to 15 days (depending on the number of samples)**

17. Acquire images of DIV20-21 neurons immunostained for the proteins of interest using a STED inverted microscope (Abberior Expert Line 775 nm, Abberior Instruments GmbH, Göttingen, Germany).

**CRITICAL:** Before acquiring images from the sample, ensure that the excitation and the depletion lasers foci of the STED microscope are precisely aligned using fluorescent beads of known sizes.

18. Select a Region of Interest (ROI) and acquire a confocal and a super resolved STED image of the same ROI with a sampling of 0.015 μm.
19. This system uses a pulsed depletion laser at 775 nm and two pulsed excitation lasers at 561 nm and 640 nm. Adjust the respective total power for excitation-561 nm, excitation-640 nm, and STED-775 nm to 70%, 50% and 40% of their total power, respectively. The settings employed for STED imaging are shown in Figures 1A–1D.

**Figure 1:**
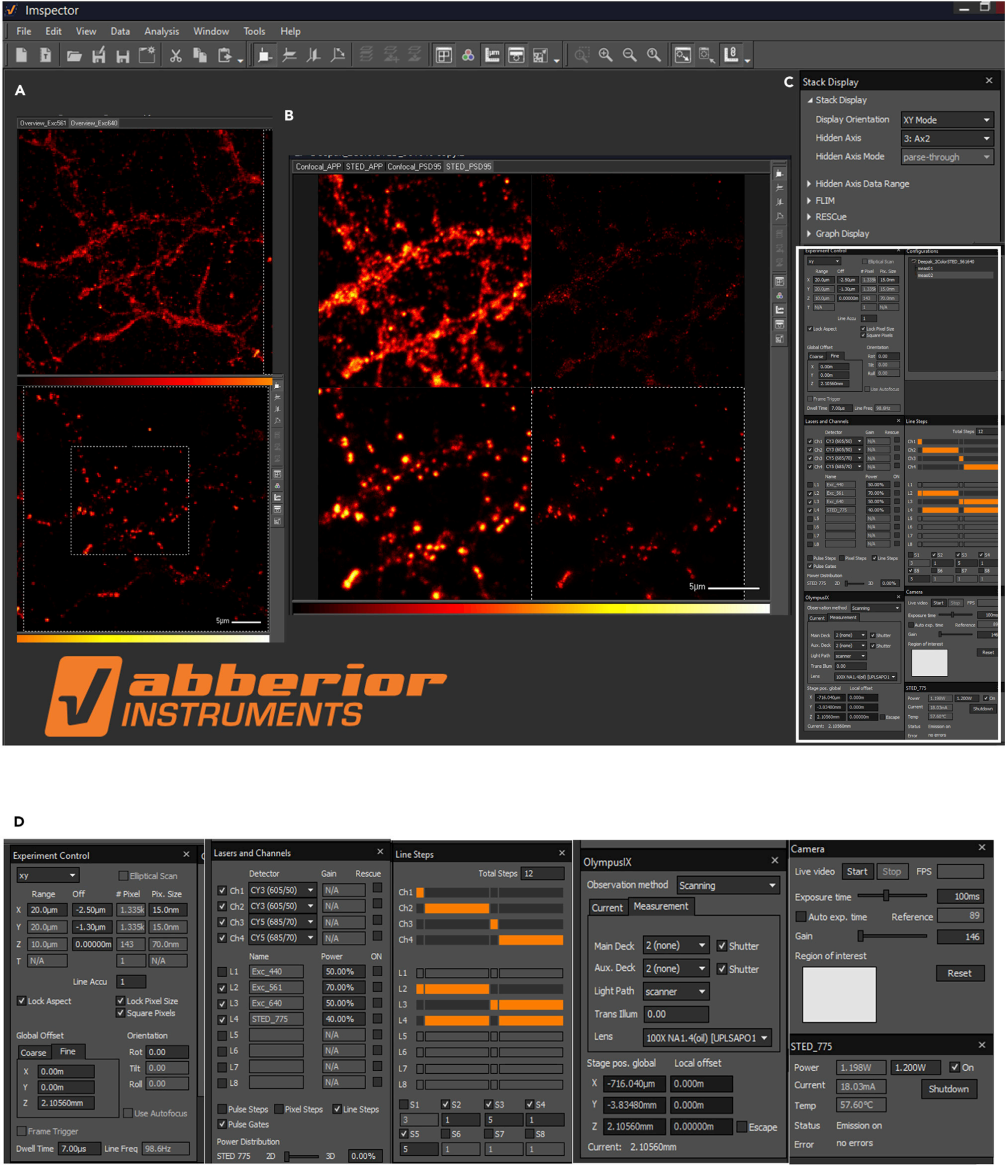
Confocal and STED imaging using an Abberior STED inverted microscope (A) ROI selection for imaging. (B) Confocal and STED image acquisition for APP (Alexa fluor 594) and PSD95 (Abberior star red dye). (C) A display of parameters used for imaging. (D) Magnified inset of the region indicated in (C).
20. Save images with a bit depth of 16 bits as MSR files (.msr).

## Key resources table

**Table:** 

REAGENT or RESOURCE	SOURCE	IDENTIFIER
**Antibodies**		

Rabbit polyclonal anti-APP-CT	Calbiochem/Millipore	Cat#171610; RRID: AB_211444
Mouse monoclonal anti-PSD95	Thermo Scientific	Cat#MA1-046; RRID: AB_2092361
Guinea pig polyclonal anti-Shank2	Synaptic Systems	Cat#162204; RRID: AB_2619861
Goat anti-rabbit Alexa Fluor 594	Life Technologies	Cat#A11037; RRID: AB_2534095
Goat anti-mouse Alexa Fluor 488	Life Technologies	Cat#A11029; RRID: AB_2534088
Goat anti-guinea pig Alexa Fluor 555	Life Technologies	Cat#A21435; RRID: AB_2535856
Goat anti-mouse Abberior STAR RED	Abberior	Cat#2-0002-011-2; RRID: AB_2810982

**Chemicals, peptides, and recombinant proteins**		

Paraformaldehyde	Merck	Cat#8187151000
Sucrose	Merck	Cat#61839805001730
Sodium chloride (NaCl)	Merck	Cat#1.93206.5021
Potassium chloride (KCl)	Sigma	Cat#P5405-500G
di-Sodium hydrogen phosphate (Na_2_HPO_4_)	Merck	Cat#1.93609.0521
Potassium dihydrogen phosphate (KH_2_PO_4_)	Merck	Cat#1.93205.0521
Glycine	Sisco Research Laboratories	Cat#66327
Triton X-100	Sisco Research Laboratories	Cat#64518
Bovine serum albumin (BSA)	Sisco Research Laboratories	Cat#85171
Sodium hydroxide	Alfa Aesar	Cat#A16037
EDTA, disodium	Merck	Cat#E5134
Tris-HCl	Merck	Cat#T3253
Prolong diamond antifade mountant	Life Technologies	Cat#P36961

**Experimental models: Organisms/strains**		

Mouse: C57BL/6	The Jackson Laboratory	RRID: IMSR_JAX:000664
Mouse: APPswe/PS1ΔE9	The Jackson Laboratory	RRID: MMRRC_034832-JAX

**Oligonucleotides**		

Primer: Prp Fwd: CCTCTTTGTGACTATGTGGACTGATGTCGG	(Jankowsky et al., 2004)	N/A
Primer: Prp Rev: GTGGATAACCCCTCCCCCAGCCTAGACC	(Jankowsky et al., 2004)	N/A
Primer: APP Fwd: CCGAGATCTCTGAAGTGAAGATGGATG	(Jankowsky et al., 2004)	N/A

**Software and algorithms**		

Imspector	Abberior	https://imspector.abberior-instruments.com/
ImageJ (Fiji) v1.52p	ImageJ	https://imagej.net/
MetaMorph v7.10.1.181	Molecular Devices	https://www.moleculardevices.com
PALMTracer plugin	(Izeddin et al., 2012; Kechkar et al., 2013; Nair et al., 2013)	N/A
Microsoft Excel	Microsoft	https://www.microsoft.com
GraphPad Prism v7.04	GraphPad	https://www.graphpad.com/scientific-software/prism/

**Other**		

Cover glasses	Marienfeld	Cat#0117580
12-Well cell culture plate	Thermo Scientific	Cat#150628
Petri dish	Tarsons	Cat#460091
Parafilm	Tarsons	Cat#380020
Microslides	Corning	Cat#2948-75**×**25
STED inverted microscope (expert line 775 nm)	Abberior	https://abberior-instruments.com/
Zeiss LSM 880	Zeiss	https://www.zeiss.com/

## Materials and equipment

The list of reagents needed are as follows:

**Table:** 10× PBS

Reagent	Final concentration	Amount
NaCl	1.4 M	80 g
KCl	27 mM	2 g
Na_2_HPO_4_	101 mM	14.4 g
KH_2_PO_4_	18 mM	2.4 g
Milli Q H_2_O	N/A	Up to 1 L

***Note:*** Adjust the pH to 7.4. Dispense the solution into aliquots and sterilize by autoclaving. Store at 26^°^C–28^°^C. Stable for 3–4 months.

***Alternatives:*** Commercially available ready to use PBS solutions can also be used.

**Table:** 1× PBS

Reagent	Final concentration	Amount
10× PBS	N/A	100 mL
Milli Q H_2_O	N/A	Up to 1 L

***Note:*** Dispense the solution into aliquots. Store at 26^°^C–28^°^C. Stable for 3–4 months.

**Table:** 4% Paraformaldehyde solution in PBS

Reagent	Final concentration	Amount
Paraformaldehyde	4%	40 g
1× PBS	N/A	Up to 1 L

***Note:*** Filter the solution with filter paper and adjust the pH to 6.9. Dispense the solution into aliquots. Store at 4^°^C. Stable for 1–2 months. Add sucrose, 4 g in 100 mL of solution and then use paraformaldehyde solution as a fixative.

**CRITICAL:** Paraformaldehyde is toxic and a known carcinogen. The solution should be made inside a fume hood wearing gloves and safety glasses.

**Table:** 0.1M Glycine solution in PBS

Reagent	Final concentration	Amount
Glycine	0.1M	7.5 g
1× PBS	N/A	Up to 1 L

***Note:*** Dispense the solution into aliquots. Store at 4^°^C. Stable for 1–2 months.

**Table:** 0.25% Triton X-100 solution

Reagent	Final concentration	Amount
Triton X-100	0.25%	2.5 mL
Milli Q H_2_O	N/A	Up to 1 L

***Note:*** Dispense the solution into aliquots. Store at 26^°^C–28^°^C. Stable for 6–7 months.

**Table:** 10% BSA solution in PBS

Reagent	Final concentration	Amount
BSA	10%	100 g
1× PBS	N/A	Up to 1 L

***Note:*** Dispense the solution into aliquots. Store at 4^°^C. Stable for 2–3 months.

**Table:** 3% BSA solution in PBS

Reagent	Final concentration	Amount
10%BSA	3%	300 mL
1× PBS	N/A	Up to 1 L

***Note:*** Dispense the solution into aliquots. Store at 4^°^C. Stable for 2–3 months.

**Table:** 50× Tail lysis buffer

Reagent	Final concentration	Amount
NaOH	1.25 M	50 g
EDTA (disodium)	10 mM	3.36 g
Milli Q H_2_O	N/A	Up to 1 L

***Note:*** Adjust pH to 12.0. Dispense the solution into aliquots and sterilize by autoclaving. Store at 4^°^C. Stable for 12 months to 18 months. For 1× working solution, dilute 50× stock 1:50 in Milli-Q water and store at 4^°^C. Stable for 12 months.

**Table:** 50× Tail neutralization buffer

Reagent	Final concentration	Amount
Tris-HCl	2M	315.20 g
Milli Q H_2_O	N/A	Up to 1 L

***Note:*** Adjust pH to 5.0. Dispense the solution into aliquots and sterilize by autoclaving. Store at 4^°^C. Stable for 18 months. For 1× working solution, dilute 50× stock 1:50 in Milli-Q water and store at 4^°^C. Stable for 12 months.

## Step-by-step method details

### Semiautomated detection of dendritic compartments and functional zones of an excitatory synapse

**Timing: several hours (depending on the number of images)**

This is the first step of the image processing on confocal and STED images. This step describes a semiautomated segmentation procedure to perform micron-nanoscale morphometry analysis. We have employed an object-based strategy to segment images into several objects of interest. The protocol outlines the analysis on confocal images to detect pre/post/perisynapses and on their corresponding sub diffraction STED super resolution images to distinguish functional zones of an excitatory synapse (CAZ/PSD/EZ) from rest of the neuronal processes.

1. Save each MSR image file into TIFF file format using ImageJ (Fiji) (https://imagej.net/)(Schindelin et al., 2012; Schneider et al., 2012).
2. Load TIFF images into MetaMorph software (Molecular Devices) (Figure 2A).

**Figure 2:**
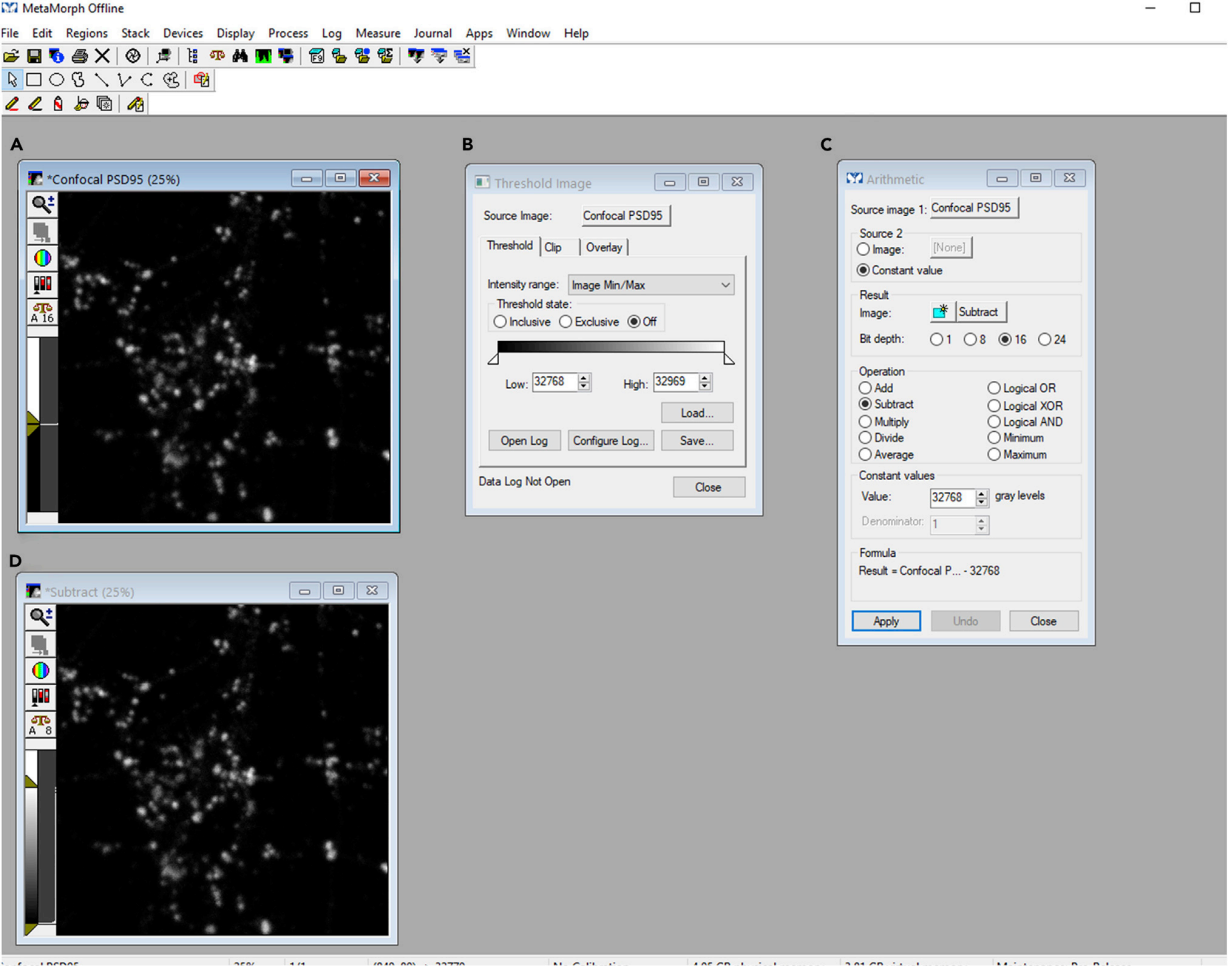
Subtract operation on an image opened in MetaMorph software using threshold and arithmetic function (A) Confocal PSD95 image opened in MetaMorph. (B) Threshold image function to find low and high limits of the image. (C) Using the arithmetic function to subtract a set gray level from confocal PSD95 image. (D) An output subtracted image.
3. Use threshold image function to verify the dynamic range of the grayscale values in the image indicated as low and high limits of the image (Figure 2B).
4. Use the arithmetic function (process menu) to subtract a set gray value (lowest gray value as obtained in step 3) from the source image. Under the process menu, select arithmetic. An arithmetic dialog box will appear. In the arithmetic box, the source image 1 will be the image of interest, and under source 2, select constant value. Select operation as subtract and value as 32768 gray levels. 32768 is the lower limit of the threshold as determined in step 3. Apply and obtain the subtracted image (Figures 2C and 2D). Save the generated image for corrected background values as a new TIFF file.
5. Reload background corrected TIFF images into MetaMorph. Under the edit menu, select image info and check if the image is calibrated (Figure 3A and 3B). If the image is not calibrated, X and Y calibration will be displayed in pixels (Figure 3B). To calibrate distances, from the measure menu, use calibrate distances command. Input X and Y calibration in real units in accordance with the sampling of images during acquisition and then select apply (Figure 3C). Here, images were obtained with a sampling of 15 nm; therefore, X and Y calibration is taken as 0.015 μm (Figure 3C).

**Figure 3:**
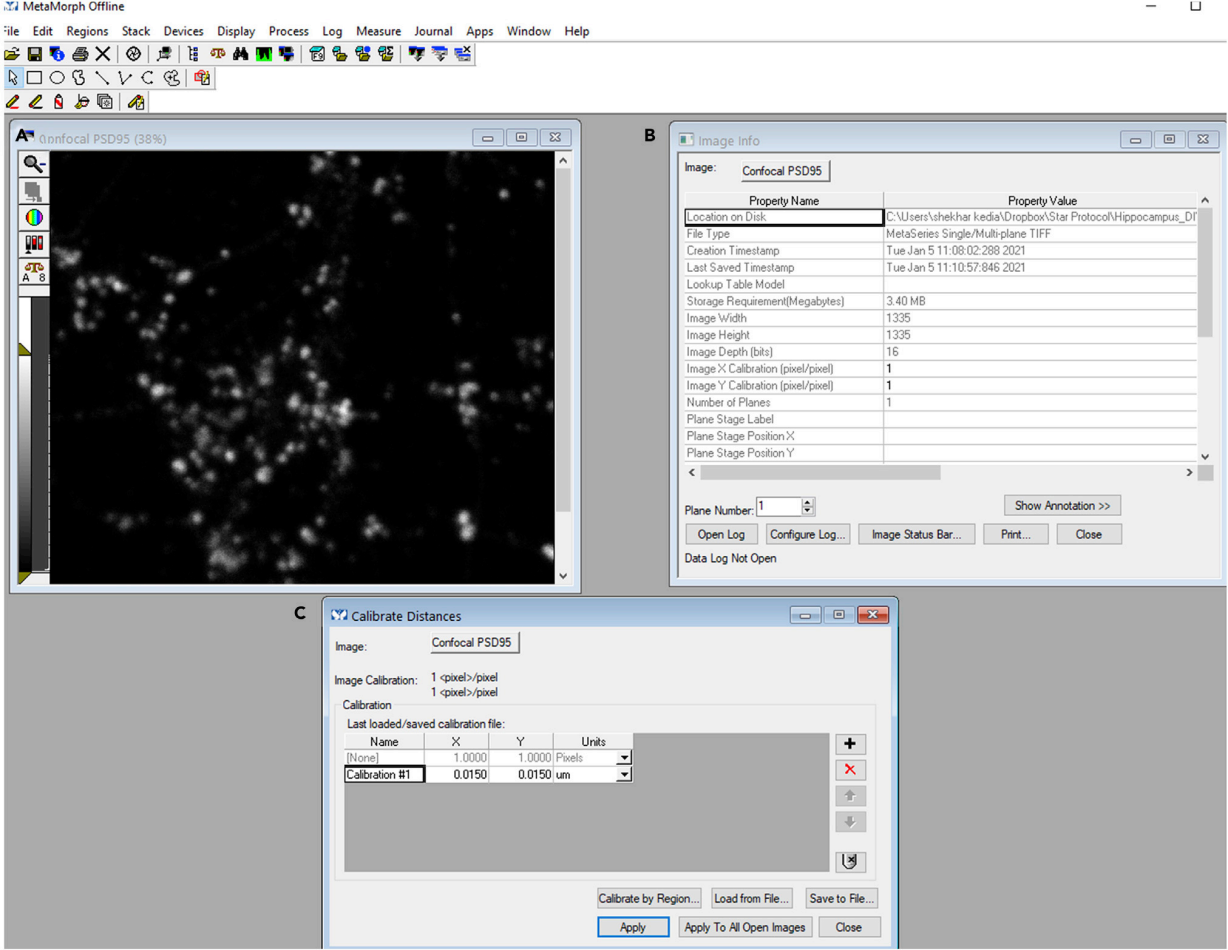
Calibrate distances command to calibrate distances in real units rather than pixels in MetaMorph software (A) Uncalibrated confocal PSD95 image revealed by image information in (B). (B) Image information command to perform a check on the calibration of the loaded image. (C) Calibrate distances command to calibrate the confocal PSD95 image.

***Note:*** To save time, all the images where analysis is to be performed could also be calibrated in a single step. To do this, open all the images at once in MetaMorph. Instead of selecting apply in the previous step, select apply to all open images from the calibrate distances dialog box (Figure 3C). It will calibrate all the images in a single click.

**Figure 4:**
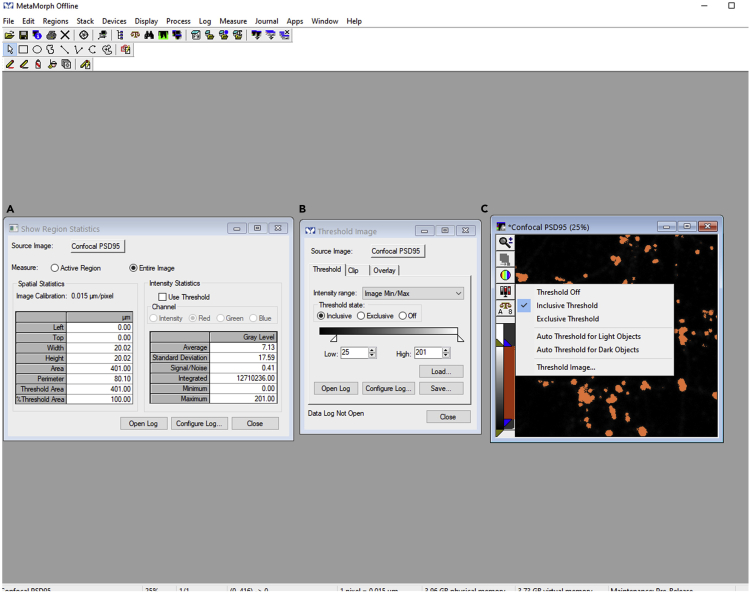
Image thresholding using inclusive threshold and show region statistics command in MetaMorph software (A) Show region statistics command to measure average and standard deviation of the intensity for the entire confocal PSD95 image. The set of objects meeting specified criteria are marked in green color. (B) Inclusive threshold function for thresholding confocal PSD95 image. (C) A thresholded confocal PSD95 image.
  c. Within this image, select objects with intensity higher than average intensity added to the standard deviation of the intensity of the entire image. Here, the average intensity plus standard deviation of the intensity is 25 gray levels (rounding off to the nearest integer) (Figure 4A).
  d. Using the threshold image function, select the threshold state to be inclusive and input the low threshold value to 25 gray levels, which is the average intensity plus standard deviation of the intensity value for this image. The pixels which are above 25 gray levels are assigned pseudocolour as orange to identify selected puncta (Figures 4B and 4C).
  e. Filter synaptic puncta using several morphological filters like length, breadth, and area which characterizes the synapse morphometry (Harris and Stevens, 1989; Harris and Weinberg, 2012). It is necessary for the image to be thresholded before performing this analysis. Make sure that your image is already thresholded (steps 1–4).
  f. Use measure menu to open Integrated Morphometry Analysis (IMA) dialog box (Figures 5A and 5B). Select the source image as confocal PSD95 and select specific object classifier filters like length >= 0.06 μm, breadth >= 0.045 μm and area as >= 0.006 μm^2^ as limit 1 (Figure 5B). Select measure and then create an object mask (Figure 5B). This step filters the set of objects which meet the specified quantitative criteria and are marked in green color (Figure 5A). On the contrary, the objects which are excluded from the analysis maintain the thresholded image orange color (Figure 5A). Generate a binary IMA object mask as an output image (Figure 5D). In this binary image, objects of interest are set to white color (maximum) and the objects which are excluded during IMA filtering are set to black color (minimum) (Figure 5D).
    **CRITICAL:** Ensure that only the required parameters like length, breadth, and area are selected with a check mark in the filter menu from the IMA module dialog box. While all other parameters remain unchecked in the filter menu.
    ***Optional:*** Depending upon the kind of the research question being investigated, in cases where it is required to quantify the morphological and biophysical parameters of the synaptic puncta itself, the object data could also be exported and saved as an excel file. For this, select the object data tab from IMA module. For logging data into an excel file, select open log. This opens an object log dialog box, then log measurements to Dynamic Data Exchange (DDE) on the object log dialog box. It will export the data to an excel file by logging the data to the file after pressing F9 (Figures 5C, 5E, and 5F).
    ***Note:*** Apart from measuring individual synaptic puncta parameters through IMA as an object log, IMA can also quantify a summary of the information as a summary log. For the later, select the summary tab from the IMA module. The data could be similarly exported to an excel file, as described in the previous step.
    ***Note:*** IMA module can perform analysis of various types of morphological and biophysical parameters related to the area, dimension, and intensity. According to the user question and need; these parameters could be customised by opting select measurement tab from the IMA module. It will open the select measurements dialog box where the useful parameters could be selected as per the user requirement.

6. Repeat steps 1–5 for all the images.

7. Perform semiautomated detection of synaptic puncta as follows:
  a. Load background corrected and calibrated (steps 1–5) confocal image of the protein of interest in MetaMorph. In this case, a postsynaptic marker PSD95 confocal image is opened in MetaMorph. PSD95 is a marker for postsynaptic density, and PSD95 confocal image could be used to segment synaptic puncta from the rest of the dendrite.
  b. Object-based segmentation of synaptic puncta involves thresholding the image and segmenting objects based on the thresholding. In this context, from measure menu, select show region statistics command to measure the average intensity and the standard deviation of the intensity for the entire confocal PSD95 image. For instance, here, the average intensity is 7.13 gray levels, and the standard deviation of the intensity is 17.59 gray levels. Image calibration to 0.015 μm/pixel can also be observed in the show region statistics menu (Figure 4A).
    **CRITICAL:** Make sure in the show region statistics dialog box, the active region is not selected.

**Figure 5:**
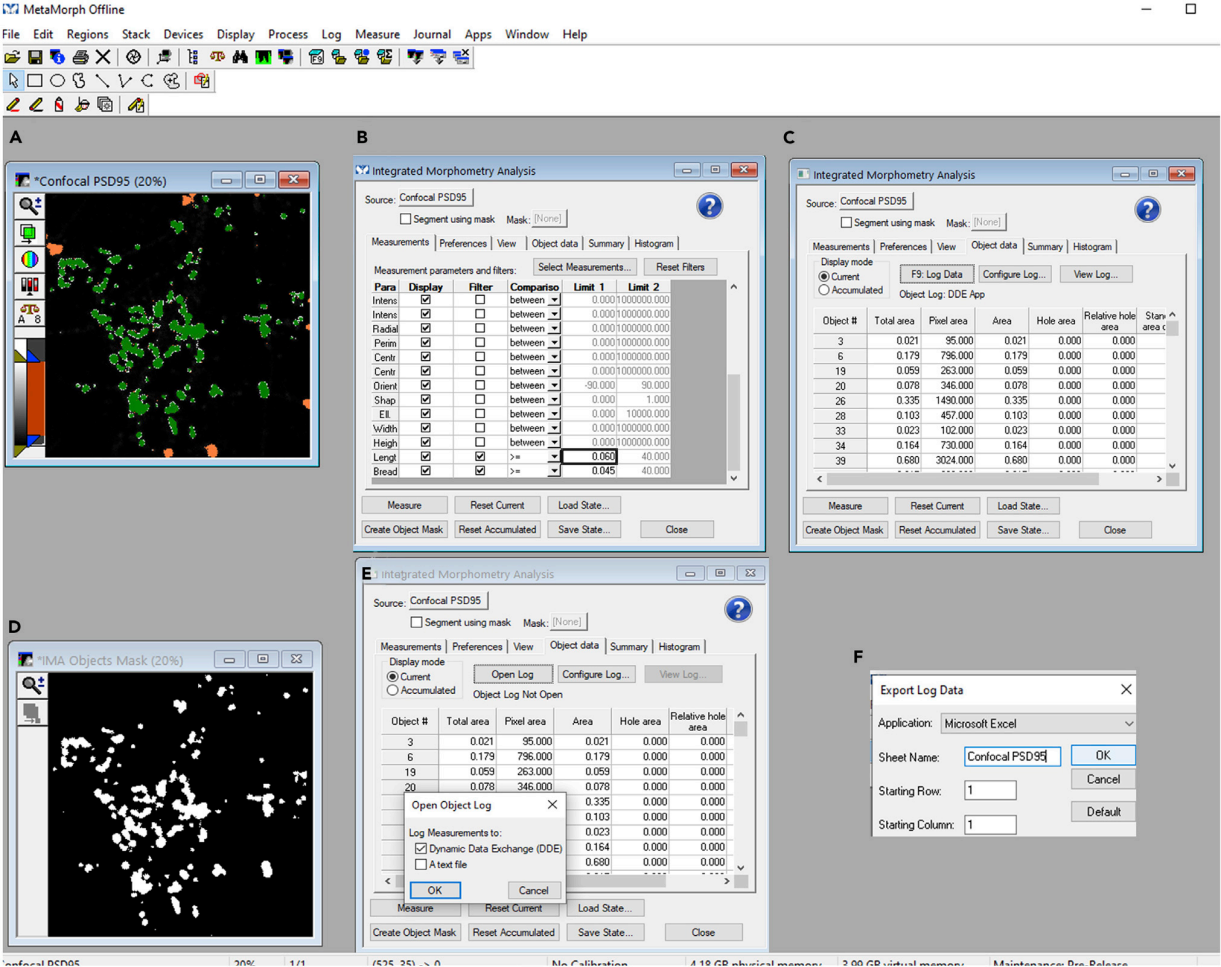
Object-based segmentation of synaptic puncta using IMA in MetaMorph software (A) A source image as confocal PSD95 image selected for specific objects based on length, breadth, and area filters. (B) A display of filtering parameters in IMA. (C) Object data for the synaptic puncta obtained through IMA. (D) A binary IMA object mask created as an output image. (E and F) Dynamic data exchange command for exporting the object data on postsynaptic puncta to an excel file.
  g. Create ROIs on the filtered objects (refer above to step f). Draw ROIs around all filtered objects or synaptic puncta. From the region menu, select create regions around objects and the source image selected should be the IMA objects mask created in step g (Figure 6A). The boundary of the regions drawn around each object is marked with a yellow color (Figure 6B).

**Figure 6:**
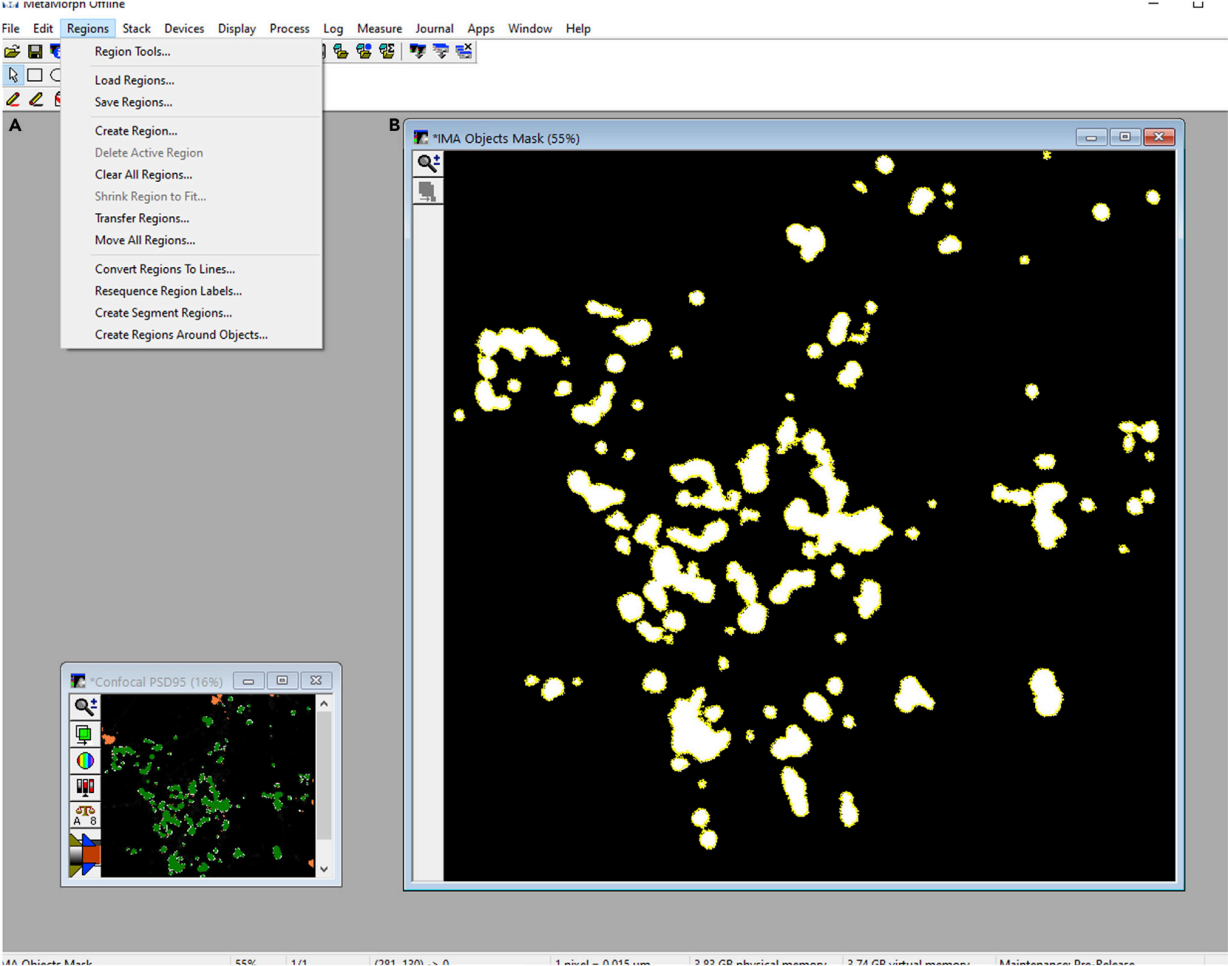
Creating ROIs on the filtered objects in MetaMorph software (A) Region menu displaying several options like creating regions around objects or transferring regions from one image to another. (B) Creating ROIs around the objects filtered with IMA object mask as the source image. The ROIs are marked in yellow color.
  h. Save the ROIs drawn in the previous step to a region file. From regions menu, select save regions. This function will save all ROIs to a region (.rgn) file. The multiple ROIs saved to a region file can be reloaded to the image of interest, for instance, IMA object mask through load regions function of the region menu.
    ***Note:*** The ROIs can also be transferred from one image to a different image using transfer regions function of the region menu. ROIs can also be cleared from an image using clear all regions function from region menu (Figure 6A).
    ***Note:*** Here, the detection of synaptic puncta is shown for the postsynaptic puncta filtered from the rest of the dendrite from confocal PSD95 images. Similar analysis (steps 1–7) can be performed on epifluorescence or confocal images of markers for different functional zones of an excitatory synapse like presynapse, postsynapse or perisynapse to identify pre/post/perisynaptic puncta.

**Figure 7:**
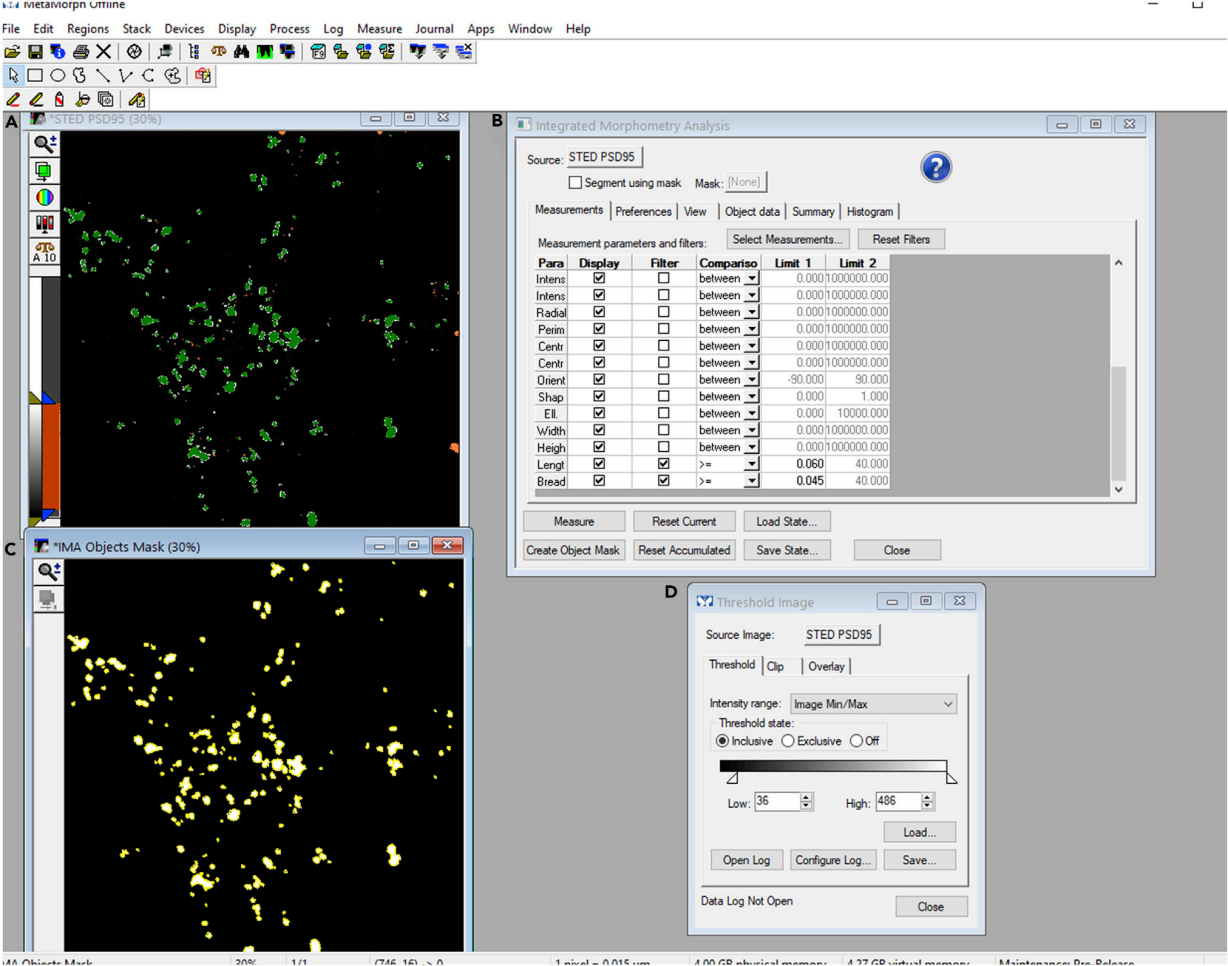
Semiautomated detection of functional zones (PSD) of an excitatory synapse using MetaMorph software (A and C) STED PSD95 image and a corresponding binary IMA object mask with regions created around objects segmented as PSD regions using IMA. The filtered PSDs are marked in green while the region boundaries are shown in yellow. (B and D) The IMA and thresholding parameters employed for filtering PSDs from STED PSD95 image as a source image.
  b. Perform object-based segmentation as described previously in steps 7b–7d. However, in contrast, to step 7d, select objects with intensity higher than average intensity added to two times the standard deviation of the intensity of the entire image. Here, average intensity plus two times the standard deviation of the intensity is 36 gray levels (rounding off to the nearest integer) (Figures 7B–7D).
  c. Filter objects through IMA similar to steps 7e and 7f and generate a binary IMA object mask as an output image (Figures 7B–7D).
    ***Note:*** As mentioned previously in step 7f (optional), if desired, the PSD quantitative data can also be exported to an excel file to reveal information about the morphological and biophysical traits of PSD.
  d. Create ROIs and save all ROIs to a region file similar to steps 7g and 7h (Figure 7C).
    ***Note:*** Here, the detection of synaptic functional zone is shown for the postsynaptic density filtered from STED PSD95 images. Similar analysis (step 8) can be performed on STED images of markers for different zones of the synapse to identify cytomatrix at the active zone (CAZ), postsynaptic density (PSD) and endocytic zone (EZ) functional compartments of an excitatory pre/post/perisynapse from rest of the neuronal processes (Kedia et al., 2021; Venkatesan et al., 2020).

8. Perform semiautomated detection of functional zones of an excitatory synapse as follows:
  a. Load background corrected and calibrated (steps 1–5) STED image of the protein of interest in MetaMorph. In this case, a postsynaptic marker PSD95 STED image is opened in MetaMorph. PSD95 is a marker for postsynaptic density, and PSD95 STED image could be used to segment PSD from the rest of the dendrite (Figure 7A).

### Super-resolution cluster analysis

**Timing: several hours (depending on the number of images)**

During the first step of image analysis, the synapses (pre/post/peri) or the functional zones of the synapse (CAZ/PSD/EZ) are filtered. The region files saved in the previous step denotes the detected functional compartments from the confocal/STED synaptic marker image (reference image). In this second step, STED super-resolution images (subject image) of proteins of interest (APP in this protocol) are analyzed to quantify nano-clusters of molecular aggregation. It outlines a method to quantify nanodomains of any protein in different functional compartments of a synapse from super resolution images using PALMTracer (Izeddin et al., 2012; Kechkar et al., 2013; Nair et al., 2013), a custom software integrated as a plugin of MetaMorph (Molecular Devices).

**Figure 8:**
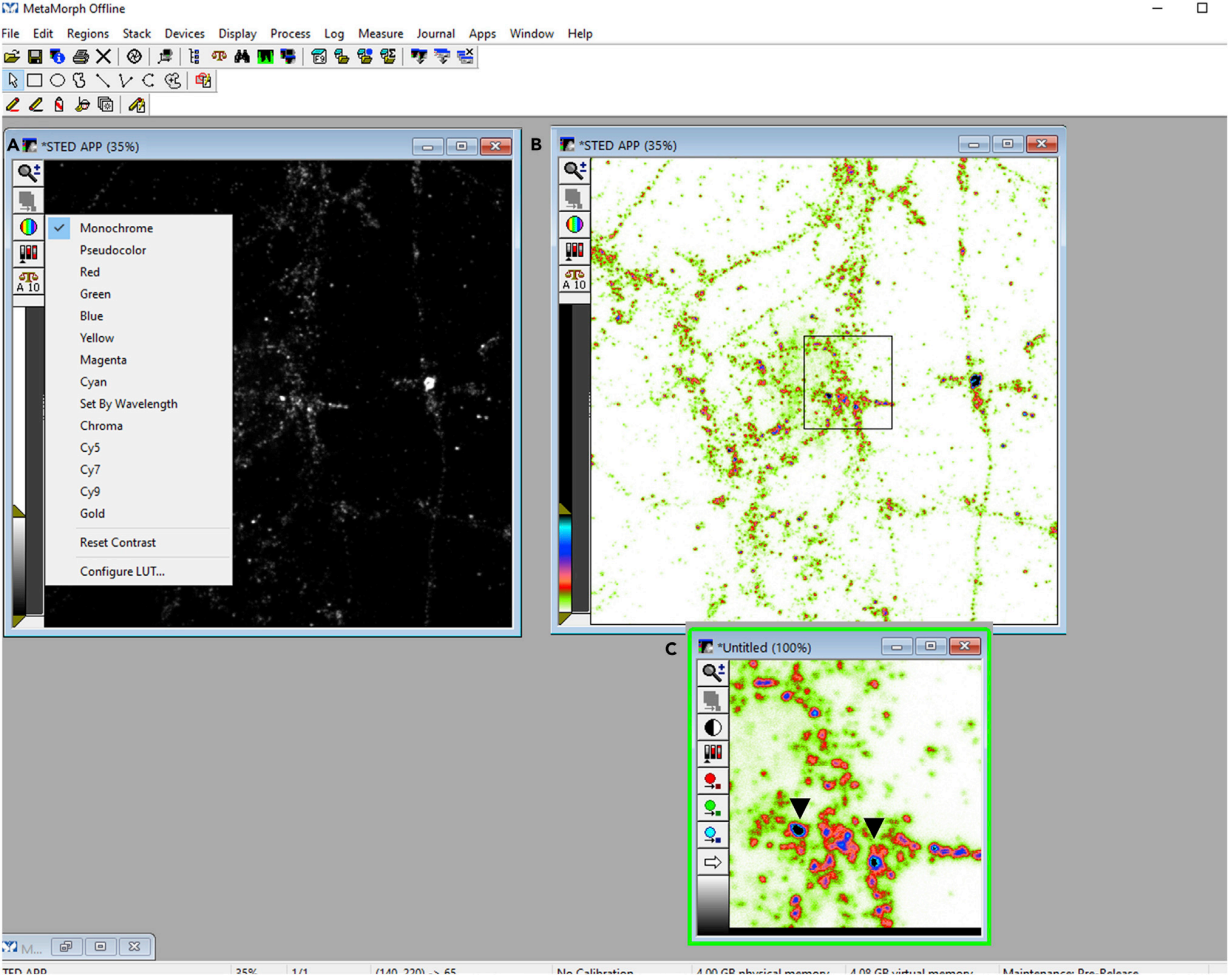
Detection of nanodomains of protein of interest in MetaMorph software (A) Super resolved image of protein of interest (APP) loaded in MetaMorph software. (B) Contrast inverted STED APP image in pseudocolor display mode. Intensity is pseudocolor coded from white (minimum) to black (maximum). (C) A magnified view of a ROI from (B). Arrows indicate domains of APP clustering to indicate region with high fluorescence intensity.

***Note:*** Perform TIFF file conversion and subtract all the subject images similarly as described in steps 1–5 in the section semiautomated detection of dendritic compartments and functional zones of an excitatory synapse.

**Figure 9:**
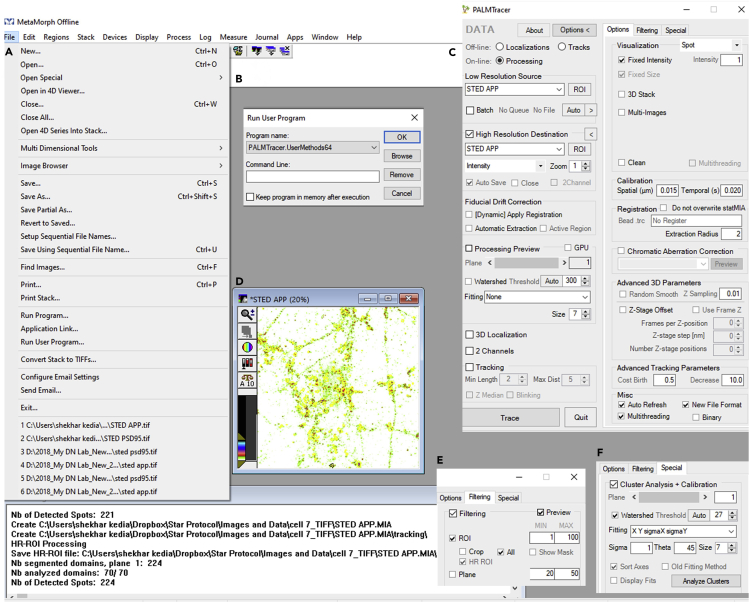
Super resolution cluster analysis of a protein of interest in synaptic sub compartments in PALMTracer plugin supported by MetaMorph software (A and B) Steps to open PALMTracer plugin running inside MetaMorph software. (C) Data and options tab of PALMTracer showing the parameters employed for cluster analysis. (D) Super resolution image of a protein of interest (STED APP) opened in MetaMorph loaded with regions from STED PSD95 image. The boundaries of the regions are indicated in yellow color. (E and F) Filtering and special tab of PALMTracer showing the parameters employed for cluster analysis.
  c. In data tab of PALMTracer, check source image selected should be STED APP (subject image), tick high resolution destination, select zoom (XY sampling) as 1 and tick autosave to save data generated automatically (Figures 9C and 9D).
  d. In options tab, input spatial calibration as 0.015 μm (sampling 15 nm during imaging). (Figure 9C).
  e. In filtering tab, tick filtering, ROI, all, and HR ROI. This step is to enable super resolution cluster analysis only in the selected ROIs (Figure 9E).
  f. In special tab, tick watershed (algorithm inbuilt in the MetaMorph program to improve the separation of closely spaced objects in an image) (Figure 9F). Select bi-dimensional Gaussian fitting for the cluster analysis as fitting XY sigma X sigma Y (Figure 9F). Select sigma for the Gaussian fit as 1, size for Gaussian fitting as 7 (see Troubleshooting) pixels and theta (free angle in degrees) as 45 degrees (Figure 9F). Tick sort axes to extract both the principal (length) as well as auxiliary axes (width) for quantifying traits of nanodomains (Figure 9F). This step performs fitting on each APP cluster that is detected as a domain. Do not change other default settings.
  g. Set threshold as auto threshold (see Troubleshooting). It is set to 27 gray levels for the STED APP image (Figure 9F).
    ***Note:*** The threshold should be empirically determined by the user for different categories of protein. Mostly, it should be either kept close to the auto threshold or to 80% of the auto threshold. However, once the threshold is fixed to either auto or 80% of auto threshold for a particular class of protein, it should be kept consistent across all the images for which cluster analysis is needed for that specific protein to avoid biasness in the results.
  h. From regions menu, select load regions tab and load STED PSD95 regions saved in step 8d on the STED APP image (Figure 9D). This step ensures that APP cluster analysis is performed only on the regions where PSD is detected.
    ***Note:*** In this protocol, as an example, we have shown the detection of APP nanodomains which are localized to the PSD. However, depending upon the markers for the functional zones of the synapse, the analysis can be extended to understand the subsynaptic compartmentalization of the proteins of interest in either synaptic puncta (pre/post/perisynapse) or different functional zones of the synapse (CAZ/PSD/EZ).
  i. Select analyze clusters tab on special menu of the PALMTracer plugin (see Troubleshooting) (Figure 9F). This step performs bi-dimensional Gaussian fitting on each nanodomain segregated to the PSD. While the domains which are not fitted are automatically excluded from the final data generated.

9. Load subject image, i.e., super resolved STED image of proteins of interest (STED APP here) in TIFF format into MetaMorph software (Molecular Devices) (Figure 8A).

10. Invert the contrast using CTRL + I command and select a pseudocolor display mode from look-up table (LUT). It will code the intensity with pseudocolor from white (minimum) to black (maximum) (Figure 8B).

11. Perform super resolution cluster analysis of proteins of interest in functional zones of an excitatory synapse as follows:
  a. Notice domains of APP clustering to indicate regions where fluorescence intensity is higher (Figure 8C).
    ***Note:*** It can be easily detected in contrast inverted pseudocolor mode where high fluorescence intensity regions are color coded.
  b. Open file menu and select run user program to open the PALMTracer plugin running inside MetaMorph (Figures 9A and 9B).
    ***Note:*** PALMTracer is a multimodal super resolution image analysis module developed as a plugin of MetaMorph (Nair et al., 2013).

## Expected outcomes

**Figure 10:**
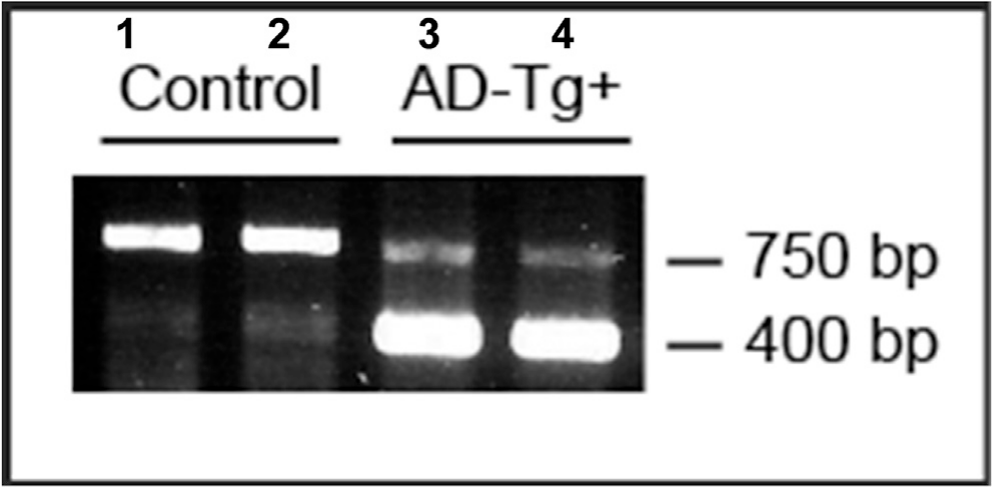
Genotyping for APP-Swe transgene DNA agarose gel showing PCR products from tail digests of 3–4 months old control (lanes 1 and 2) and APPswe/PS1ΔE9 transgenic mice (lanes 3 and 4). The control mice show amplification of 750 bp endogenous product while the transgenic mice also show the 400 bp band corresponding to the APP transgene.

Genotyping for APP-Swe transgene from tail digests of 3–4 months old control and APPswe/PS1ΔE9 transgenic mice is shown (Figure 10) The control mice show amplification of 750 bp endogenous product while the transgenic mice also show the 400 bp band corresponding to the APP transgene (Figure 10) (Jankowsky et al., 2004).

**Figure 11:**
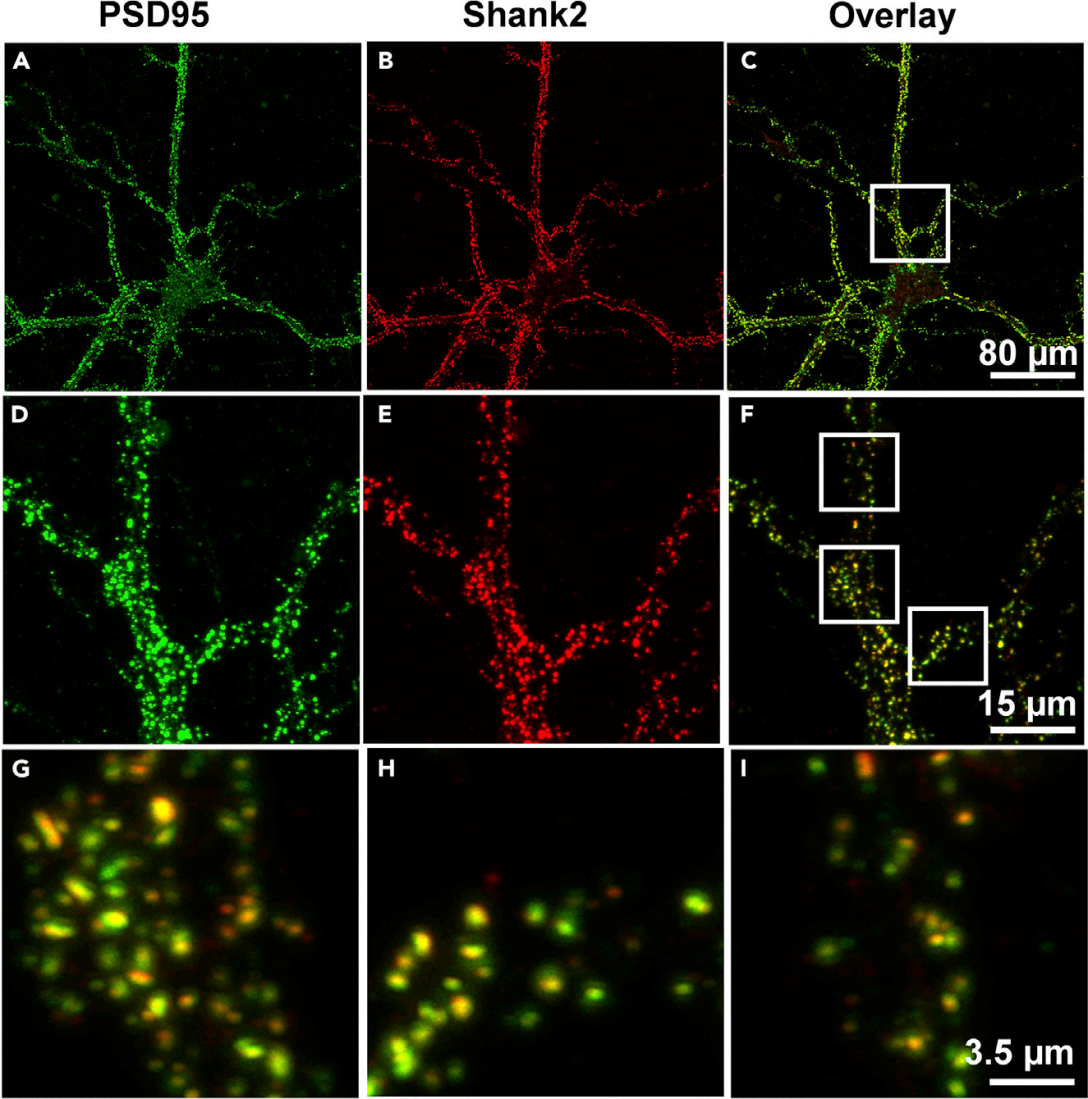
Sequential magnification depicts the punctate distribution of excitatory synapses on the dendrites of hippocampal pyramidal neurons (A–C) Indicate pyramidal neurons counter-labeled for post synaptic proteins, namely, PSD95 and Shank2 marked by Alexa 488 and Alexa 555, respectively. (C) Pseudocolor overlay of PSD95 (green) and Shank2 (red). The colocalized regions are marked in yellow. (D–F) The inset presented in (C) is represented at higher magnification with PSD95 in (D) and Shank2 in (E) with the corresponding pseudocolor overlay in (F). (G–I) Magnified images of the pseudocolor overlay marked by white regions in (F). Scale bar indicates 80 μm at (C), 15 μm at (F) and 3.5 μm at (I). Images were obtained using confocal microscopy.

Confocal imaging of two different post synaptic markers, PSD95 and Shank2 showed a discrete and punctate pattern of organization (Figures 11A–11C). The presence of both the synaptic markers in the same compartment is observed in more than 99% of the dendritic puncta (Figures 11D–11I). These observations are consistent with the results obtained in several previous studies (Kedia et al., 2020; MacGillavry et al., 2013; Nair et al., 2013; Opazo et al., 2018).

**Figure 12:**
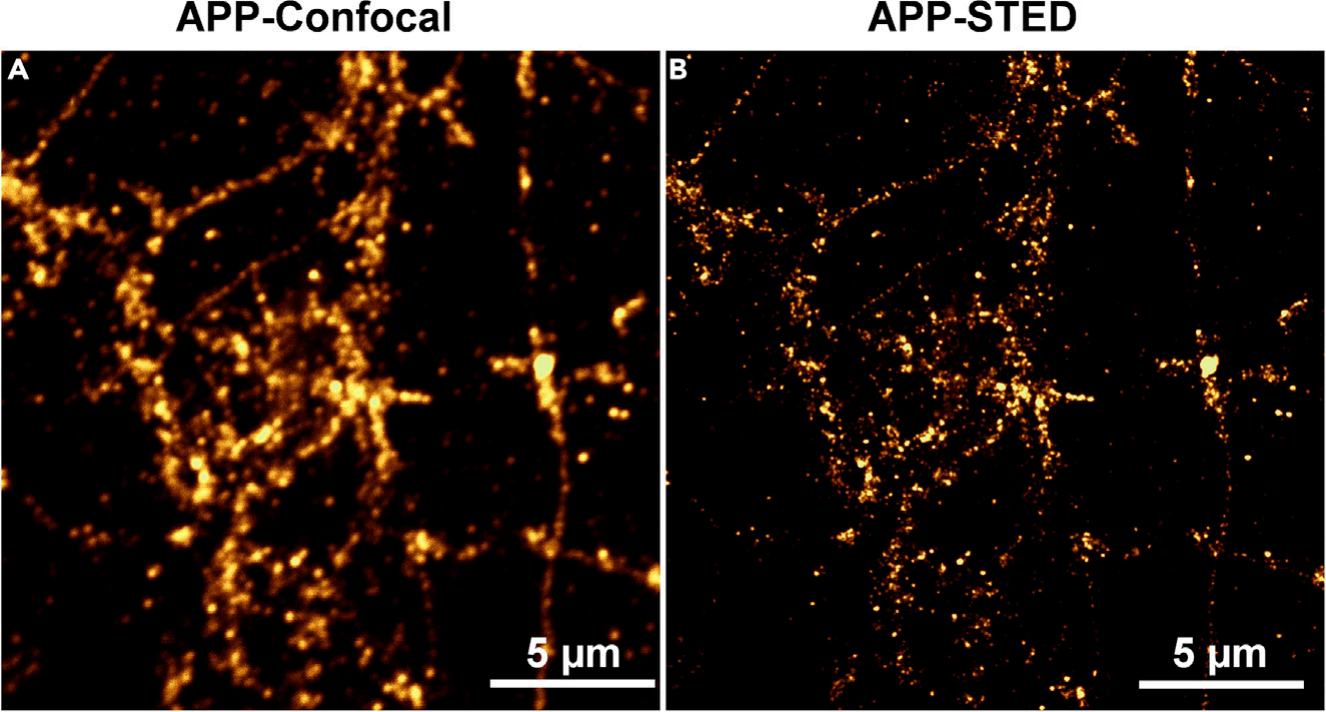
Comparison of conventional and super resolution image of APP (A) The fluorescence image of APP obtained using conventional confocal microscopy. (B) The super resolution image of APP obtained using super resolution STED microscopy. The scale bar at (A and B) indicate 5 μm.

**Figure 13:**
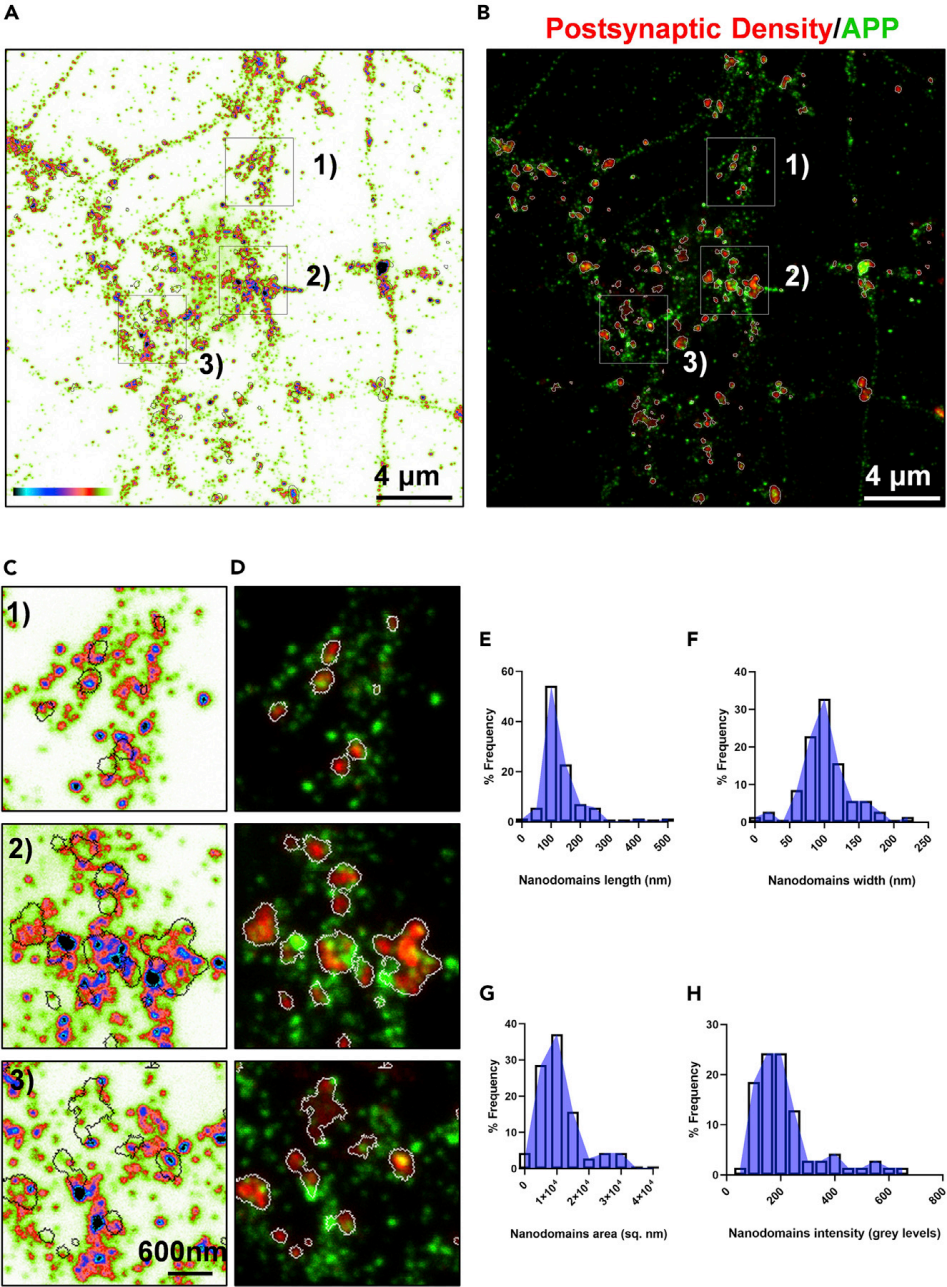
Nanoscale distribution of APP in the postsynaptic density using super resolution microscopy (A) Pseudocolor image of APP obtained using STED microscopy. The intensity is color coded from white (minimum) to black (maximum) with black contours representing the PSD regions from STED PSD95 image. (B) STED image with pseudocolor overlay of PSD95 (red) and APP (green) with white contours representing the PSD regions. (C and D) Magnified insets of the regions indicated as 1, 2 and 3 in (A and B). All the images are scaled from 0 (minimum) to 200 (maximum). The scale bar at (A and B) indicate 4 μm and at (C) indicates 600 nm. (E–H) Indicate the length, width, area, and intensity of APP nanodomains in PSD regions. n= 70 nanodomains.

A comparison between confocal image of APP and a super resolution image of APP obtained by STED microscopy in the same field of view is shown (Figures 12A and 12B). Super resolution imaging and concomitant cluster analysis revealed the presence of nanodomains of APP in neuronal processes (Figures 13A and 13C). A heterogeneous and punctate distribution of APP is observed (Figures 13A and 13C). A discrete organization of nanodomains of APP is visualized in the functional zones of the synapses like PSD (Figures 13B and 13D). These observations are in agreement with our assessments of nanodomains of APP in different functional zones of an excitatory synapse in the recent study (Kedia et al., 2020, 2021).

## Quantification and statistical analysis

Several morphological and biophysical parameters for the nanodomains like length, width, area, and intensity can be computed. For the measurement of nanodomain length multiply SigmaY with 2.3 and for width multiply SigmaX with 2.3 (Table 1). While for quantifying nanodomain area multiply half of length and half of width to 3.14 (Table 1). Biophysical parameters like the intensity of the nanodomain is also obtained (Table 1). The nanoscale distribution profile of APP clusters associated with PSD is measured and frequency of distribution is plotted (Figures 13E–13H). The graphs are plotted using GraphPad Prism v7.04. The length, width, area, and intensity of nanodomains of APP on PSD were measured to be 135.0+/-8.73 nm, 100.7+/-4.31 nm, 12204+/-1452 nm^2^, 216.80+/-15.60 gray levels. Data are shown as mean+/-SEM. These mean values are obtained from 70 nanodomains of APP on PSD from a single image taken as an example to outline this protocol.

**Table 1:** An example of cluster analysis data for APP nanodomains localized to the postsynaptic density

Serial number	ROI (PSD)	SigmaY (nm)	Length (nm)	SigmaX (nm)	Width (nm)	Area (nm^2^)	Intensity (Grey Levels)
1	128	61.56	141.588	40.862	93.98193	10445.77	161.4014
2	101	63.621	146.3283	47.984	110.3636	12677.21	228.413
3	122	43.299	99.5873	34.844	80.14209	6265.19	135.7762
4	32	49.38	113.5731	43.6	100.2796	8940.422	217.1852
5	100	44.869	103.1985	43.368	99.74619	8080.519	203.8232
6	83	98.079	225.5807	70.715	162.6434	28801.02	214.7626
7	138	47.522	109.3001	47.099	108.3278	9294.587	144.5906
8	45	38.334	88.16894	34.984	80.46421	5569.138	134.9115
9	110	15.393	35.40285	9.708	22.32847	620.536	264.7533
10	15	58.974	135.6395	42.806	98.45362	10483.04	186.9439
11	66	96.331	221.5605	66.026	151.8605	26412.34	484.5823
12	47	29.006	66.71276	25.18	57.91443	3032.951	77.51157
13	108	53.836	123.8227	35.795	82.32845	8002.393	180.6172
14	66	212.132	487.9037	82.071	188.7633	72297.19	566.0551
15	66	115.634	265.9591	46.313	106.5189	22238.78	187.3357

Sometimes, due to error in Gaussian fitting or due to the presence of fiducial markers like fluorescent beads in the sample, aberrant measurements like the size of clusters greater than the known size of the CAZ/PSD/EZ (Harris and Stevens, 1989; Harris and Weinberg, 2012; Nair et al., 2013) are also obtained. To avoid bias in the results due to such abnormal values, the nanodomains with size greater than 500 nm and intensity values above 5000 gray levels should be excluded from the analysis. The nanodomains data excluded in this manner are generally less than 1%–2% of the total.

For statistical evaluations of nanodomains data, depending upon the sample size at first the data should be tested for the normal distribution using D’Agostino-Pearson Omnibus normality test or Shapiro-Wilk normality test. Normally distributed datasets can be compared using two-tailed unpaired Student’s t-test (for two-group) or one-way analysis of variance (ANOVA) test followed by Tukey’s multiple comparison test (for multi-group). On the other hand, non-normally distributed datasets can be tested by non-parametric two-tailed Mann- Whitney test (for two-group) or Kruskal-Wallis test followed by Dunn’s multiple comparison test (for multi-group). Similar quantification paradigm for nanodomains in functional zones of the synapse and statistical analysis were used in our recent studies (Kedia et al., 2021).

## Limitations

This protocol has been used for the analysis of the nanoscale organization of molecules in different subcellular compartments. Though the technique is robust, there are a few limitations where the protocol would yield suboptimal results.

First, it is very important to calibrate the concentration of antibodies and have knowledge on the number of fluorophores conjugated to the secondary antibody. Both these parameters could affect the threshold of signaling or generation of pseudo clusters when the density of molecules is very sparse.

Second, an improperly calibrated STED system and suboptimal sampling could give rise to images where the resolution is deteriorated, and the analysis will not yield robust outcomes.

Third, the geometrical orientation of synapses could at times produce hotspots of local changes in intensity or clusters with altered shape factors. It could be eliminated by comparing synapses with similar morphology and orientation.

## Troubleshooting

### Problem 1

Higher background fluorescence intensity or low signal to noise ratio (immunocytochemistry, step 6).

### Potential solution

The stronger background fluorescence intensity could be due to non-optimal primary or secondary antibody concentration or inefficient blocking or washing. The primary or secondary antibody concentration should be decreased. The incubation time for blocking and washing can be increased. Sometimes, secondary antibodies can also contribute to the background signal. To test this, perform the procedure without primary antibodies. If performing co-immunostaining for more than one protein of interest, spectral bleed-through because of overlapping emission spectra of secondary antibodies should be avoided. Further, unreacted aldehydes can also increase background fluorescence; therefore, quenching of unreacted aldehydes with 0.1 M glycine should be performed after fixation.

### Problem 2

Very low or no fluorescence signal (immunocytochemistry, step 10).

### Potential solution

If very low or no fluorescence is detected, then primary or secondary antibody concentration or incubation time should be increased. The primary antibody incubation can also be performed for 12–14 h in a humidified chamber at 4^°^C. However, the fixation or permeabilization procedures can also mask the antigen, impacting the immunodetection of proteins of interest by primary antibodies. In that case, increasing the concentration or incubation time would not be a suitable solution. Instead, alternate fixatives or permeabilization reagents should be tried. Apart from this, the primary and secondary antibodies compatibilities should also be checked. It is necessary for the secondary antibody to be raised against the same host species as the primary antibody. Finally, the copy number of proteins of interest also influences the fluorescence signal generated. If the protein is present in a very low copy number, the fluorescence signal would be low. A positive control with overexpression analysis to ectopically express the protein of interest should be used.

### Problem 3

The size of the nanodomains detected for the known proteins not matching with the data already available in the literature (super-resolution cluster analysis, step 11f).

### Potential solution

**Figure 14:**
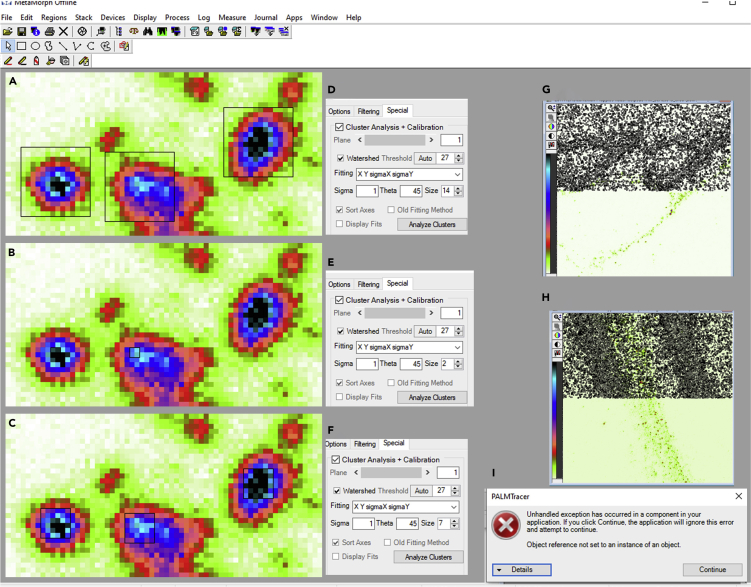
Troubleshooting PALMTracer super resolution cluster analysis (A–C) Pseudocolor image of APP obtained using STED microscopy loaded in MetaMorph software. The intensity is color coded from white (minimum) to black (maximum). (D–F) Different ROI sizes opted for the Gaussian fitting of the clusters in the special tab of PALMTracer plugin supported by MetaMorph software. The ROI size in (D) is 14, in (E) is 2 and in (F) is 7. The ROIs are indicated by black contours in (A–C). (G and H) Pseudocolor STED image of a protein of interest with an image depth of 8 bits loaded in the MetaMorph software and analyzed for clusters through PALMTracer plugin. The detected nanodomain regions are indicated in black. (I) Error message by PALMTracer during super resolution cluster analysis.

The nanodomain analysis through PALMTracer is heavily dependent on parameters defined by the user. The ROI size of the square opted for the Gaussian fitting is a very critical parameter which the user should empirically determine. An ambiguous increase or decrease in ROI size will affect the output leading to bias in the analysis. The size should be determined in a way that it selects mostly the central maxima of the intensity of the cluster (Figures 14A–14F). We also suggest that the analysis should be performed initially for the proteins whose sizes are known in the literature allowing user to calibrate the ROI size and then moving to the proteins of interest. Once the optimal ROI is determined, it should be kept consistent across all the images. Although, in our experience, a square of the size of 7 pixels (15 nm XY sampling) works well for the cluster analysis on images obtained through STED microscopy.

### Problem 4

After performing the auto threshold for cluster analysis in the PALMTracer, the nanodomains are not detected properly. In this scenario, the regions where nanodomains are visually apparent are not detected positive for nanodomains. On the contrary, the aberrant regions are identified across image as positive for nanodomains by the software (Figures 14G and 14H; super-resolution cluster analysis, step 11g).

### Potential solution

Such a drastic selection of nanodomains (Figures 14G and 14H) by the PALMTracer usually happens when the image is not suitable to be analyzed by it. This error can be due to dynamic range of images being very small (less than 8 bits). PALMTracer usually handles images with higher dynamic range in the grayscale values much better. In this scenario, the images can still be analyzed after converting it to 16 bits.

### Problem 5

Error messages during cluster analysis by PALMTracer (super-resolution cluster analysis, step 11i).

### Potential solution

Sometimes during cluster analysis by PALMTracer, a user might get several error messages like “unhandled exception has occurred” etc. (Figure 14I). These error messages can occur several times during an image analysis signifying a poor signal to noise ratio in the image due to high background intensity. Most of the time, these images could be due to suboptimal staining because of the aspecificity of antibodies arising from degradation, inadequate preparation of reagents, improper handling of the sample or erroneous aggregation. The images with higher background noise result in aberrant selection of several ROIs, making cluster analysis through PALMTracer difficult and, therefore, resulting in errors. Such images should be deemed inappropriate for the analysis and should be discarded.

## Resource availability

### Lead contact

Further information and requests for resources and reagents should be directed to and will be fulfilled by the lead contact and corresponding author, Dr. Deepak Nair at deepak@iisc.ac.in.

### Materials availability

This study did not generate any new unique reagents.

### Data and code availability

All software used in this study is listed in the key resources table. This study did not generate any additional data sets other than provided in the manuscript. Thumbnails for the Graphical abstract were prepared using Biorender.com.

## References

[bib1] Beaudoin G.M.J., Lee S.-H., Singh D., Yuan Y., Ng Y.-G., Reichardt L.F., Arikkath J. (2012). Culturing pyramidal neurons from the early postnatal mouse hippocampus and cortex. Nat. Protoc..

[bib2] Harris K.M., Weinberg R.J. (2012). Ultrastructure of synapses in the mammalian brain. Cold Spring Harb. Perspect. Biol..

[bib3] Harris M., Stevens J.K. (1989). Dendritic spines of CA1 pyramidal cells in the rat hippocampus : serial electron microscopy with reference to their biophysical characteristics. J. Neurosci..

[bib4] Izeddin I., Boulanger J., Racine V., Specht C.G., Kechkar a., Nair D., Triller a., Choquet D., Dahan M., Sibarita J.B. (2012). Wavelet analysis for single molecule localization microscopy. Opt. Express.

[bib5] Jankowsky J.L., Fadale D.J., Anderson J., Xu G.M., Gonzales V., Jenkins N.A., Copeland N.G., Lee M.K., Younkin L.H., Wagner S.L. (2004). Mutant presenilins specifically elevate the levels of the 42 residue β-amyloid peptide in vivo: Evidence for augmentation of a 42-specific γ secretase. Hum. Mol. Genet..

[bib6] Kechkar A., Nair D., Heilemann M., Choquet D., Sibarita J.B. (2013). Real-time analysis and visualization for single-molecule based super-resolution microscopy. PLoS One.

[bib7] Kedia S., Ramakrishna P., Netrakanti P.R., Jose M., Sibarita J.B., Nadkarni S., Nair D. (2020). Real-time nanoscale organization of amyloid precursor protein. Nanoscale.

[bib8] Kedia S., Ramakrishna P., Netrakanti P.R., Singh N., Sisodia S.S., Jose M., Kumar S., Mahadevan A., Ramanan N., Nadkarni S. (2021). Alteration in synaptic nanoscale organization dictates amyloidogenic processing in Alzheimer’s disease. IScience.

[bib9] MacGillavry H.D., Song Y., Raghavachari S., Blanpied T.A. (2013). Nanoscale scaffolding domains within the postsynaptic density concentrate synaptic ampa receptors. Neuron.

[bib10] Nair D., Hosy E., Petersen J.D., Constals A., Giannone G., Choquet D., Sibarita J.-B. (2013). Super-resolution imaging reveals that AMPA receptors inside synapses are dynamically organized in nanodomains regulated by PSD95. J. Neurosci..

[bib11] Opazo P., Viana da Silva S., Carta M., Breillat C., Coultrap S.J., Grillo-Bosch D., Sainlos M., Coussen F., Bayer K.U., Mulle C. (2018). CaMKII metaplasticity drives aβ oligomer-mediated synaptotoxicity. Cell Rep..

[bib12] Schindelin J., Arganda-Carreras I., Frise E., Kaynig V., Longair M., Pietzsch T., Preibisch S., Rueden C., Saalfeld S., Schmid B. (2012). Fiji: An open-source platform for biological-image analysis. Nat. Methods.

[bib13] Schneider C.A., Rasband W.S., Eliceiri K.W. (2012). NIH Image to ImageJ: 25 years of image analysis. Nat. Methods.

[bib14] Venkatesan S., Subramaniam S., Rajeev P., Chopra Y., Jose M., Nair D. (2020). Differential scaling of synaptic molecules within functional zones of an excitatory synapse during homeostatic plasticity. ENeuro.

